# Targeting Annexin A1 as a Druggable Player to Enhance the Anti-Tumor Role of Honokiol in Colon Cancer through Autophagic Pathway

**DOI:** 10.3390/ph16010070

**Published:** 2023-01-01

**Authors:** Xi Wang, Gang Shao, Xiangyu Hong, Yue Shi, Yiting Zheng, Yucheng Yu, Caiyun Fu

**Affiliations:** 1 Department of Oncology, No.903 Hospital of PLA Joint Logistic Support Force, Hangzhou 310013, China; 2 Zhejiang Provincial Key Laboratory of Silkworm Bioreactor and Biomedicine, College of Life Sciences and Medicine, Zhejiang Sci-Tech University, Hangzhou 310018, China; 3 Key Laboratory of Preclinical Study for New Drugs of Gansu Province, School of Basic Medical Sciences & Research Unit of Peptide Science, Chinese Academy of Medical Sciences, 2019RU066, Lanzhou University, Lanzhou 730000, China

**Keywords:** annexin A1, colon cancer, hyperproliferation, honokiol, autophagy, reactive oxygen species, drug resistance

## Abstract

Colon cancer is one of the most common digestive tract malignancies, having the second highest mortality rate among all tumors, with a five-year survival of advanced patients of only 10%. Efficient, targeted drugs are still lacking in treating colon cancer, so it is urgent to explore novel druggable targets. Here, we demonstrated that annexin A1 (ANXA1) was overexpressed in tumors of 50% of colon cancer patients, and ANXA1 overexpression was significantly negatively correlated with the poor prognosis of colon cancer. ANXA1 promoted the abnormal proliferation of colon cancer cells in vitro and in vivo by regulating the cell cycle, while the knockdown of ANXA1 almost totally inhibited the growth of colon cancer cells in vivo. Furthermore, ANXA1 antagonized the autophagic death of honokiol in colon cancer cells via stabilizing mitochondrial reactive oxygen species. Based on these results, we speculated that ANXA1 might be a druggable target to control colon cancer and overcome drug resistance.

## 1. Introduction

Colon cancer is one of the most common digestive tract malignancies, with the third incidence rate after breast cancer and lung cancer [1]. The mortality rate has increased year by year and has surpassed liver cancer and gastric cancer to reach second place [1,2]. Despite the treatment methods for colon cancer that have been developed in the past few decades, it has little effect on the prognosis; therefore, it is still urgent to explore novel targets and potent therapeutic drugs.

Annexin A1 (ANXA1), a Ca^2+^-regulated phospholipid-dependent and membrane-binding protein, has been identified at the beginning as an inhibitor of phospholipase A2 to regulate inflammation [3,4]. Recent studies have shown that ANXA1 plays an important role in tumors, participating in the processes of tumorigenesis [5], growth [6], invasion [7], migration [8], and drug resistance [9]. However, the function of ANXA1 seems to be distinct in different or even the same tumor [10]. In melanoma and pancreatic cancer, ANXA1 is increased and involved in the regulation of proliferation [11,12]. Low expression of ANXA1 has been confirmed in breast cancer tumor tissues, but there is still overexpressing ANXA1 in poorly differentiated, invasive tumors [13], therefore, it is crucial to further clarify the roles and mechanisms between ANXA1 and malignancies. Until now, the function of ANXA1 in colon cancer, especially the relationship between ANXA1 expression and its response to drug treatment, has hardly been reported.

Honokiol is an active ingredient of the traditional Chinese herb “*Houpo*”; it is found in the *Magnolia* tree and used to treat “stagnation of *qi*” [14,15]. Copious studies have verified the antioxidative and anticarcinogenic properties of polyphenols [16]. Honokiol, as one kind of polyphenol, can play an anti-tumor role through various mechanisms [17], such as antiangiogenesis [18], endoplasmic reticulum stress [19], and targeting mitochondria [20]. In colon cancer, Notch, Hippo, TGF-β, and multiple signaling pathways were considered to involve in honokiol-induced cell death [21,22,23,24]. However, tumors are less sensitive to honokiol than some other diseases, and the mechanisms that cause this difference remain unclear [25,26,27].

Here, we uncovered that ANXA1 promotes colon cancer cell proliferation by regulating the cell cycle, and targeting ANXA1 can enhance the anti-tumor role of honokiol by regulating the autophagic signaling pathway through mitochondrial reactive oxygen species (mtROS).

## 2. Results

### 2.1. ANXA1 Is Overexpressed in Colon Cancer Tissues Related to a Significant Negative Correlation with the Poor Prognosis of Colon Cancer

**Figure 1:**
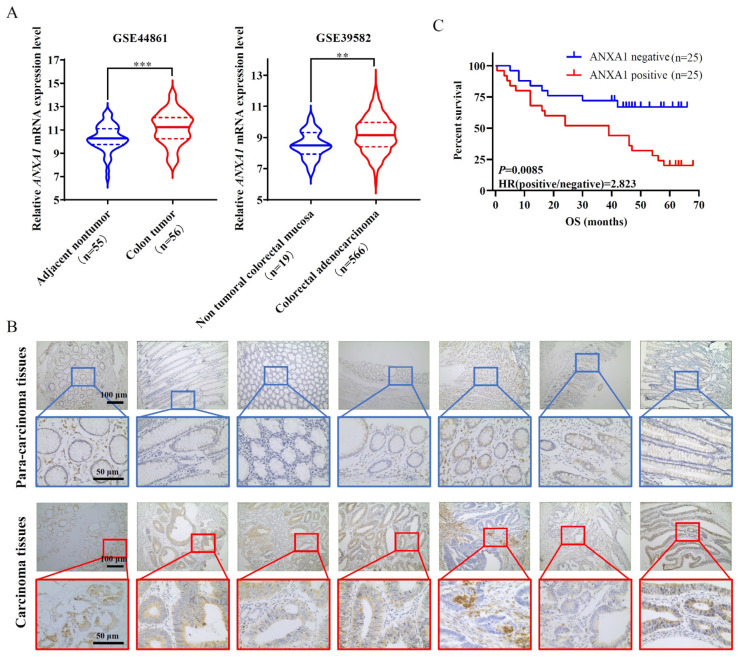
ANXA1 is overexpressed in colon cancer tissues, related to a significant negative correlation with the poor prognosis of colon cancer. (**A**) *ANXA1* mRNA expression levels in the colon or colorectal tumors and adjacent nontumor tissues from GSE44861 and GSE39582 datasets. Student’s *t*-test, ** *p* < 0.01, *** *p* < 0.001. (**B**) Representative immunostaining of ANXA1 in colon carcinoma and para-carcinoma tissues. Scale bars are indicated in the images. (**C**) Kaplan–Meier survival curves stratified by ANXA1 expression. Log-rank test.

In the beginning, we searched the database and found that the mRNA expression of ANXA1 was elevated markedly in colon tumors compared to adjacent tissues or non-tumoral mucosa (Figure 1A), suggesting the potential role of ANXA1 in the pathogenesis of colon cancer. To explore the pathological significance of ANXA1 in colon cancer, we successively recruited 50 colon cancer patients, of which 25 patients (50%) showed positive expression of ANXA1 for their tumors via immunohistochemistry (Supplementary Table S1). Concretely, ANXA1 appeared to be overexpressed in the carcinoma tissues but not in the para-carcinoma tissues (Figure 1B). Moreover, the patients with positive ANXA1 expression showed significantly lower overall survival compared to those with negative ANXA1 expression (Figure 1C).

### 2.2. ANXA1 Promotes the Hyperproliferation of Colon Cancer Cells

**Figure 2:**
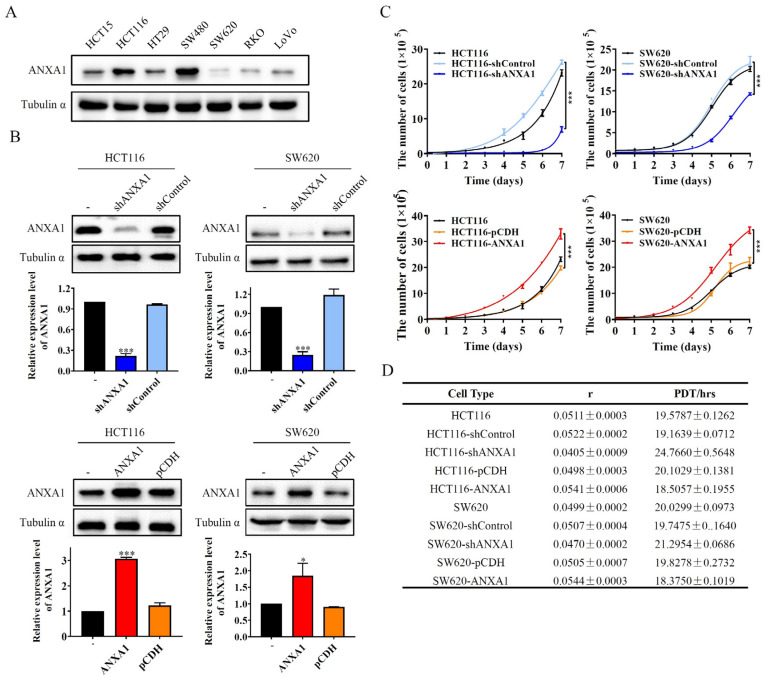
ANXA1 promotes the hyperproliferation of colon cancer cells. (**A**) Immunoblotting images of ANXA1 expression level in colon cancer cell lines. (**B**) Immunoblotting images of ANXA1 expression level of constructed ANXA1 knockdown and overexpressed cell lines. (**C**) The cell proliferation curve of the constructed cell lines within 7 days. (**D**) The multiplication rate (r) and population doubling times (PDT) of the constructed cell lines. Student’s *t*-test, * *p* < 0.05, *** *p* < 0.001.

To investigate the effect of ANXA1 on colon cancer cell function, we first detected the expression levels of ANXA1 in seven colon cancer cell lines. Among these cell lines, ANXA1 was higher expressed in two cell lines—HCT116 and SW480—and lower expressed in three cell lines—SW620, RKO, and LoVo (Figure 2A). Then we designed the plasmids of ANXA1 over-expressing or knockdown, and then we transfected HCT116 and SW620 cells to stably up- or down-regulate the expression levels of ANXA1 (Figure 2B). In both HCT116 and SW620 cell lines, knockdown of ANXA1 significantly inhibited cell proliferation rates, while overexpression of ANXA1 significantly promoted cell proliferation (Figure 2C,D).

### 2.3. ANXA1 Regulates the Cell Cycle of Colon Cancer

**Figure 3:**
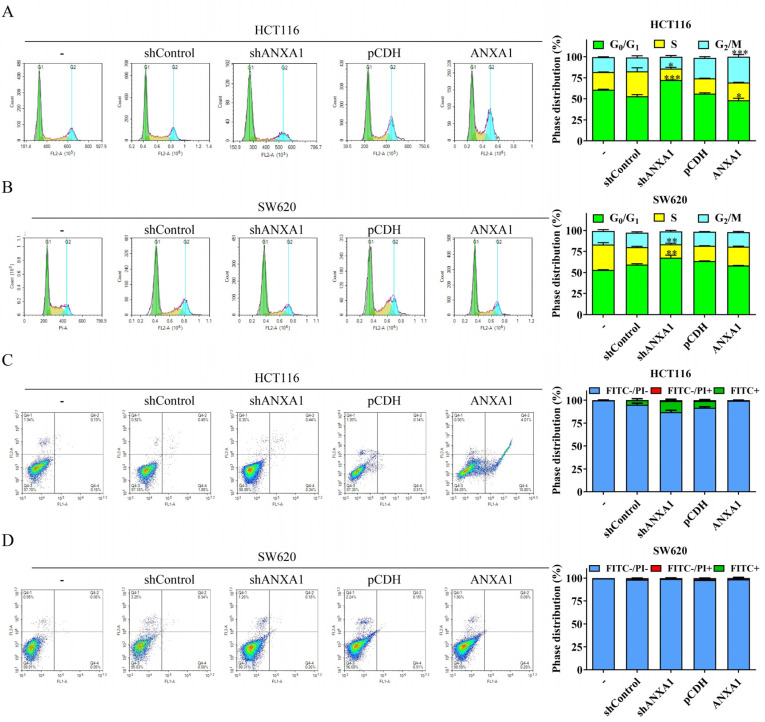
ANXA1 regulates the cell cycle of colon cancer. (**A**,**B**) Representative cell cycle distribution diagrams and statistical charts of HCT116 (**A**) and SW620 (**B**) cells. (**C**,**D**) Representative annexin V-FITC/PI staining distribution diagrams and statistical charts of HCT116 (**C**) and SW620 (**D**) cells. Student’s *t*-test, * *p* < 0.05, ** *p* < 0.01, *** *p* < 0.001.

Next, we found that ANXA1 regulates the cycle distribution of colon cancer cells. Specifically, knockdown of the expression of ANXA1 significantly reduced the proportion of cells in the S phase, as well as increased the proportion of cells in G_0_/G_1_ phase in both the HCT116 and SW620 cell lines (Figure 3A,B). In contrast, overexpression of ANXA1 increased the proportion of cells in the S phase and reduced the proportion in G_0_/G_1_ phase in both HCT116 and SW620 cells (Figure 3A,B). Alternatively, the overexpression of ANXA1 appeared to have an effect on the proportion of cells in the G_2_/M phase. Knockdown of ANXA1 reduced the proportion of G_2_/M phase cells in both HCT116 and SW620 cells, while the proportion of G_2_/M phase of HCT116 cells was significantly increased after ANXA1 overexpression (Figure 3A,B). However, neither knockdown nor overexpression of ANXA1 induced apoptosis in HCT116 and SW620 cells (Figure 3C,D).

### 2.4. ANXA1 Promotes Colon Tumor Growth In Vivo

**Figure 4:**
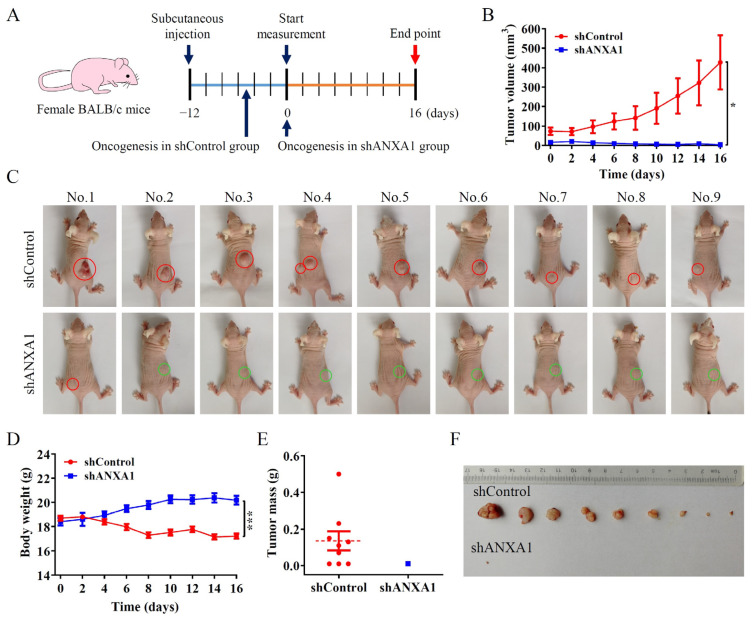
ANXA1 promotes colon tumor growth in vivo. (**A**) Schematic diagram of the course of the mice experiment. (**B**) Statistical chart of tumor volume in the mice, *n* = 9. (**C**) Representative images of the mice at day 16. The red circles indicate the tumors, and the green circles indicate the injection sites without tumors. (**D**) Statistical chart of body weight of the mice, *n* = 9. (**E**) Statistical chart of the tumor weight difference between control and shANXA1 groups. (**F**) Representative images of the tumors isolated from control and shANXA1 groups. Student’s *t*-test, * *p* < 0.05, *** *p* < 0.001.

To further investigate the role of ANXA1 protein in colon cancer growth in vivo, we constructed tumor-bearing nude mice models by subcutaneous injection of the HCT116-shControl cells and the HCT116-shANXA1 cells in BALB/c mice. The body weight and tumor volume of mice in the HCT116-shControl group and the HCT116-shANXA1 group were measured once every two days, beginning on the 12th day after injection and continuing for another 16 days (Figure 4A). After 12 days of injection, tumors developed in all mice of HCT116-shControl group with an average volume of 73.84 ± 19.00 mm^3^, while only 5 mice in HCT116-shANXA1 group developed a tumor with an average volume of 16.48 ± 2.178 mm^3^ (Figure 4B,C). In addition, mice in HCT116-shANXA1 group weighed significantly higher than those in the control group from day 6 (Figure 4D). On day 16, the experiment ended and the tumors were stripped; only one mouse in the HCT116-shANXA1 group still had a small tumor (Figure 4E,F). Thus, our results showed that HCT116 cells with ANXA1 knockdown had a lower rate of tumor formation, and the resultant tumor grew slowly and tended to subside easily in vivo compared with parent cells.

### 2.5. ANXA1 Antagonizes Autophagy Induced by Honokiol in Colon Cancer Cells

**Figure 5:**
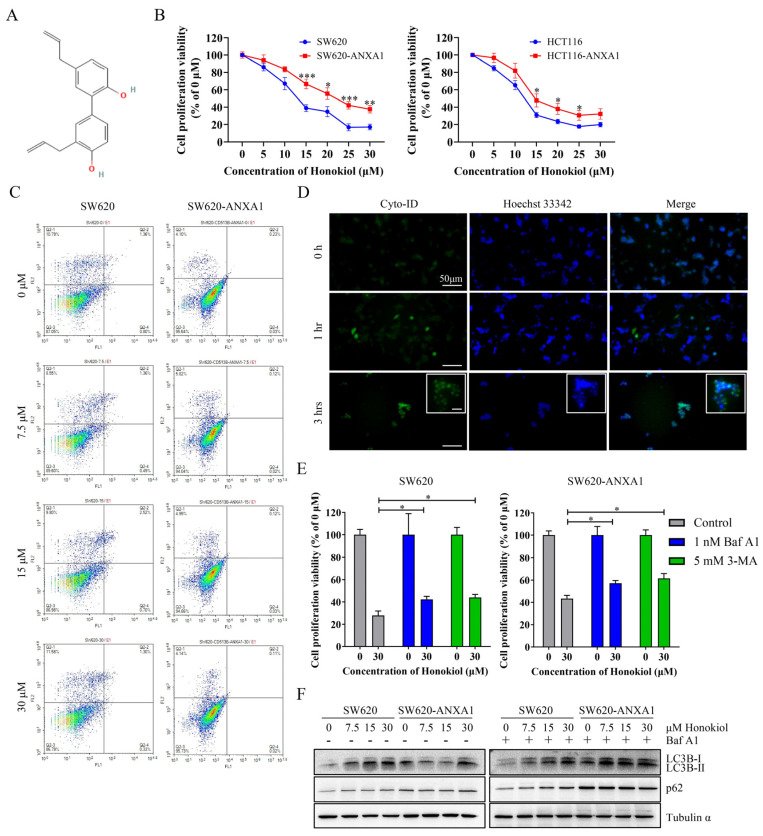
ANXA1 antagonizes autophagy induced by honokiol in colon cancer cells. (**A**) The structural formula of honokiol was obtained from PubChem (CID: 72303). (**B**) Proliferation viability of parents and ANXA1 overexpressed HCT116 and SW620 cells treated by honokiol with concentration gradient for 24 h. (**C**) Representative annexin V-FITC/PI staining distribution diagrams of SW620 and SW620-ANXA1 cells treated by honokiol with concentration gradient for 24 h. (**D**) Representative fluorescent staining images of autophagy induced by 30 μM honokiol after pretreatment with 1 nM bafilomycin A1. Scale bar, 50 μm. (**E**) The effect of bafilomycin A1 and 3-methyladenine on the inhibitory effect of honokiol on cell proliferation. (**F**) Immunoblotting images of the expression of autophagy marker proteins in SW620 cells after honokiol treatment for 24 h. Whether 1 nM bafilomycin A1 was pretreated is indicated in the figure. Student’s *t*-test, * *p* < 0.05, ** *p* < 0.01, *** *p* < 0.001.

Honokiol (3,5′-diallyl-4,2′-dihydroxybiphenyl, Figure 5A) has been reported to play an anticancer role in a variety of tumors [17]. Here we found that honokiol inhibited the proliferation of HCT116 and SW620 cells more potently than that of ANXA1 overexpression cells (Figure 5B). Honokiol was a potent anticancer agent through the activation of apoptosis in ovarian carcinoma cells [28] and leukemia cells [29], as well as through activation of both apoptosis and autophagy in osteosarcoma cells [30], neuroblastoma cells [31], and lung cancer cells [32]. Our results showed that the mode of death induced by honokiol in SW620 cells appeared to be neither apoptosis nor necrosis (Figure 5C) and may be autophagy according to our fluorescent imaging results (Figure 5D). Moreover, both 3-methyladenine (3-MA), an inhibitor of autophagosome formation, and bafilomycin A1 (Baf A1), an inhibitor of autophagosome-lysosome fusion, significantly alleviated honokiol-induced cell death in SW620 cells and SW620-ANXA1 cells (Figure 5E). The results of Western blotting also revealed that LC3B conversion (LC3B-I to LC3B-II) was up-regulated in SW620 cells after honokiol treatment, which could reflect the intensity of autophagy (Figure 5F). After Baf A1 treatment with the result of blocking the degradation of autophagosome-lysosome, it was obviously observed that p62 (also known as SQSTM1) was elevated with the increase of honokiol concentration in SW620 cells, suggesting that honokiol promoted the formation of autophagosomes, although the change was inconspicuous after honokiol treatment without Baf A1 (Figure 5F). In contrast, such changes in p62 expression levels appear to be absent in SW620-ANXA1 cells (Figure 5F). These results suggest that honokiol promotes autophagy in colon cancer, whereas overexpression of ANXA1 was resistant to the honokiol effect.

### 2.6. ANXA1 Antagonizes Autophagy Induced by Honokiol via Stabilizing mtROS in Colon Cancer Cells

**Figure 6:**
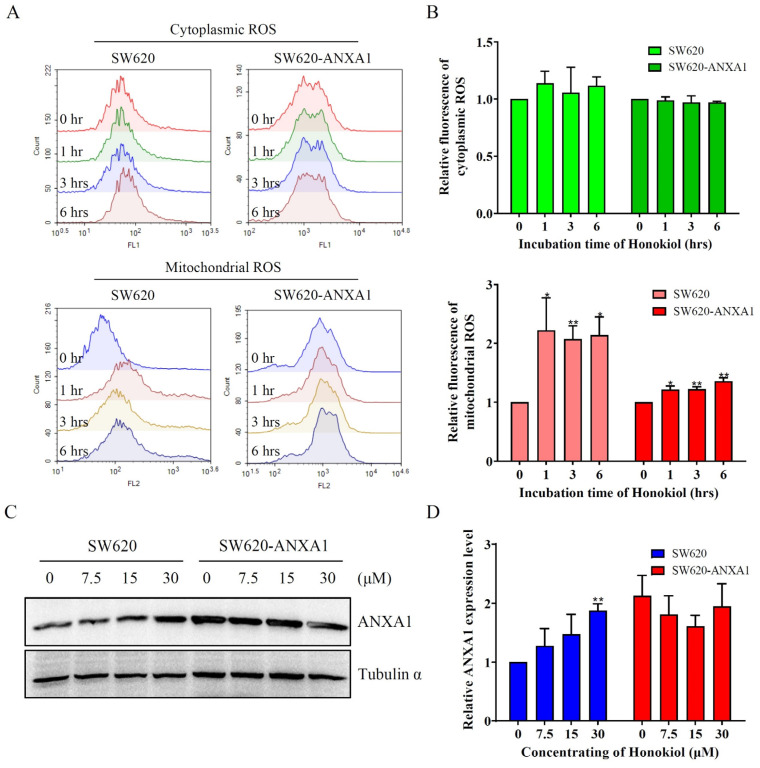
ANXA1 exerts its effects against honokiol by stabilizing mtROS in colon cancer cells. (**A**) Fluorescence signal distribution of cytoplasmic and mitochondrial reactive oxygen species of colon cells treated by 30 μM honokiol. (**B**) Statistical chart of the relative fluorescence signal of cytoplasmic and mitochondrial reactive oxygen species treated by honokiol. (**C**) Immunoblotting images of the expression level of ANXA1 in SW620 and SW620-ANXA1 cells treated by honokiol with a concentration gradient for 24 h. (**D**) Statistical chart of the relative ANXA1 expression level of SW620 and SW620-ANXA1 cells were treated with honokiol with a concentration gradient. Student’s *t*-test, * *p* < 0.05, ** *p* < 0.01.

Autophagy is activated in response to various stresses, such as increased levels of ROS [33]. Notwithstanding, honokiol did not appear to affect cytoplasmic ROS, it rapidly increased the mtROS in SW620 cells within 1 h (Figure 6A,B). This process seems to be greatly slowed down in ANXA1 over-expressed SW620 cells (Figure 6A,B). Surprisingly, honokiol itself treatment significantly increased the expression level of ANXA1 in SW620 cells (Figure 6C,D).

## 3. Discussion

Although colorectal cancer is the most common gastrointestinal malignancy, and its five-year survival rate is as high as 90% through surgery in the early stage [34], once colon cancer progresses to an advanced stage, the five-year survival rate is only about 10% [35]. For colon cancer with persistently rising morbidity and mortality, discovering new diagnostic markers and developing new therapeutic strategies are imperative. In addition to surgery, colon cancer has developed various chemotherapy drugs such as oxaliplatin, 5-fluorouracil, capecitabine, and irinotecan, as well as biological drugs such as bevacizumab, trastuzumab, pertuzumab, and cetuximab, while these antibodies targeting the VEGF and EGFR families have very limited therapeutic efficacy in the advanced colon cancer [36,37]. Although KRAS is one of the most frequently mutated genes in colon cancer, it is difficult to develop targeted drugs due to its binding to GTP and the lack of pocket structure in the active site [38,39]. For BRAF (V600E) mutated colon cancer, vemurafenib feedback activates EGFR, although it can inhibit BRAF (V600E) [40]. The complex cross-regulatory network of signaling pathways in colon cancer makes the current targeted therapy of colon cancer face a great dilemma, which prompts us to find more new targets with druggable potential and develop new targeted drugs.

ANXA1 has the potential to be a target in multiple tumors. Targeting ANXA1 abolished Treg-mediated immunosuppression in triple-negative breast cancer [41]. RRM2 modulates the anti-tumor effect of sunitinib and PD-1 blockade in renal cancer by stabilizing ANXA1 [9]. ANXA1 also binds and stabilizes EphA2 to promote nasopharyngeal carcinoma growth and metastasis [6]. Exosomal ANXA1 has been implicated in the malignant transformation of thyroid follicular epithelial cells in thyroid cancer [42]. In this study, we found that ANXA1 can also be used as a diagnostic and prognostic indicator in colon cancer. Tumor heterogeneity in colon cancer severely limits the drug response, which makes the drug not often effective. The heterogeneity is also reflected by the ANXA1 expression difference in seven colon cancer cell lines. But ANXA1 can significantly regulate the proliferation ability of both HCT116 cells with higher ANXA1 expression and SW620 cells with lower ANXA1 expression, suggesting that ANXA1 may be a universal target for colon cancer.

As a natural product drug extracted from the *Magnolia* tree, honokiol has recently been found to make an impact on various signaling pathways in tumor cells. The role of honokiol includes the induction of apoptosis and autophagy in osteosarcoma cells by ROS/ERK1/2 signaling [30], suppression of pancreatic cancer progression, and invasion by miR-101/Mcl-1 and SMAD2/3 axis [27,43], reduction of gastric tumor growth and peritoneal dissemination by inhibiting Tpl2 [44], and antagonism of doxorubicin resistance in breast cancer through miR-188-5p/FBXW7/c-Myc pathway [45]. It has also been reported that honokiol can inhibit colon cancer cell proliferation through signals such as TGF-β1/p53 [21,46], but only one paper revealed that the death mode induced by honokiol might be iron death [47]. Honokiol can also increase the sensitivity of tumor cells to cisplatin and oxaliplatin [47,48]. In this study, we demonstrated that honokiol did not cause apoptosis in colon cancer cells, but instead exerted anti-tumor effects by inducing autophagy. However, ANXA1 inhibited the autophagy and led to the accumulation of p62, which may be due to the inhibition of the autophagosome–lysosome degradation by ANXA1 [49]. Chemoresistance is a major bottleneck in colon cancer treatment, and ANXA1 is thought to regulate chemoresistance in a variety of tumors. It includes modulation of sensitivity to sunitinib and PD-1 through activation of the AKT pathway in renal cancer [9], and also generating resistance to trastuzumab through activation of AKT in breast cancer [50]. These studies suggest that ANXA1-overexpressing cells exhibiting a worse drug response to honokiol may be related to their activation of drug-resistance-related signals. Hypoxia is a common feature of the tumor microenvironment, under which the mitochondria of tumor cells regulate the balance of ROS, leading to HIF-1α accumulation, activating protective autophagy and promoting tumor cell survival [51,52]. Although Honokiol increased mitochondrial oxygen consumption rate and reduced ROS synthesis in wild-type cells, it further increased ROS production in tumor cells [30,53], which caused dysregulated ROS homeostasis and inhibition of HIF-1α, and finally induced apoptotic or autophagic death [54,55]. Our study found that ANXA1 antagonizes the anti-tumor effects of honokiol by stabilizing ROS. Interestingly, honokiol promoted the up-regulation of ANXA1 while exerting its anti-tumor role, in turn leading to drug resistance that may also be related to hypoxia induction [9,50,56]. This negative feedback mechanism is similar to the EGFR feedback activation by verofenib in colon cancer and by lomvartinib in hepatocellular carcinoma [40,57]. These negative feedback regulatory mechanisms may be key for tumor cells to activate autoprotective mechanisms to limit drug response. Thus, targeting feedback-expressed proteins such as ANXA1 is essential for maintaining and enhancing the anti-tumor effects of honokiol. In addition, inverse molecular docking protocol may contribute to the identification of potential targets of honokiol, and advanced molecular dynamics techniques may provide valuable insights into the regulatory relationship between these targets and ANXA1 [58,59,60].

In conclusion, we demonstrated that overexpression of ANXA1 in patients and cell lines of colon cancer cause cell hyperproliferation and poor prognosis, and ANXA1 is the key factor in weakening the autophagic death induced by honokiol via stabilizing mtROS in colon cancer. Thus, our study highlights ANXA1 as a promising target to develop novel therapeutic drugs to treat colon cancer, even to reverse chemotherapy resistance of colon cancer.

## 4. Materials and Methods

### 4.1. Data Mining

*ANXA1* mRNA expression data of colon tumors and adjacent nontumor tissues were obtained in GSE44861 and GSE39582 dataset by reporter 201012_at from the GEO database (accessed on 25 November 2022 at https://www.ncbi.nlm.nih.gov/geo/).

### 4.2. Cell Culture and Drug Treatment

The culture conditions of HCT116 and SW620 cell lines followed the method as we previously described [61]. The cells expressing plasmid were cultured in a medium containing puromycin (Solarbio, Beijing, China). Honokiol (MedChemExpress, Trenton, NJ, USA) and bafilomycin A1 (MedChemExpress, Trenton, NJ, USA) were prepared at 10 mM in DMSO (NCM Biotech, Suzhou, China) and stored at −80 °C. 3-methyladenine (MedChemExpress, Trenton, NJ, USA) was weighed and prepared at 5 mM with culture medium when it will be used, then filtered by a 0.22 μm needle filter.

### 4.3. Plasmid Construction and Cell Screening

Plasmid construction and preparation of lentivirus were performed according to the method of Guo et al. [62]. The small hairpin RNA of *ANXA1* was constructed by inserting the sequence (5′-GCATTCTATCAGAAGATGTAT-3′) into the pLKO.1 vector. The overexpression plasmid of ANXA1 was constructed by inserting the cDNA sequence of ANXA1 (Gene ID: 301) obtained from NCBI into the pCDH vector. The plasmids were packaged into lentiviruses in 293T cells and then infected cells, which were treated by puromycin for selecting cells stably knockdown or overexpressed ANXA1.

### 4.4. Cell Proliferation Ability Assay

Cell proliferation ability assay referred to the method as we previously described [61]. Cells from the logarithmic growth phase were seeded in 48-well plates with 50,000 per well, and the cells were treated at the indicated concentration of honokiol after 24 h, with inhibitors, treated one hour in advance. After 24 h of Honokiol treatment, cells were collected and counted by a cell counter (CountStar, Shanghai, China).

### 4.5. Autophagy Detection by Fluorescence Microscopy

According to the instruction of a CYTO-ID^®^ Autophagy Detection Kit (ENZ-51031, Enzo life sciences, Farmingdale, NY, USA), cells were seeded in 48-well plates with 50,000 per well. The cells were treated with 30 μM Honokiol for 1 and 3 h, after bafilomycin A1 treatment for one hour in advance. The medium was then removed and replaced with an assay buffer containing CYTO-ID^®^ green detection reagent and Hoechst 33,342 nuclear stain. Twenty minutes later, the fluorescent images were imaged with a microscope (Axio Vert.A1, Zeiss, Oberkochen, Germany).

### 4.6. Flow Cytometry

The sample preparation for flow cytometry was performed as we previously reported [61]. In brief, cells were seeded in 6-well plates and treated with honokiol at indicated concentration and time. For cell cycle assays, cells were collected and resuspended in 75% ethanol at −20 °C overnight, then incubated with RNaseA and propidium iodide from a cycle detection kit (Keygen Biotech, Nanjing, China). For apoptosis assays, cells were digested by trypsin without EDTA and collected, then stained with annexin V-FITC/PI reagent from an apoptosis detection kit (Vazyme, Nanjing, China). For ROS detection, cells were collected after time gradient drug treatment and incubated with cytosolic reactive oxygen probe DCFH-DA (Beyotime, Shanghai, China) or mitochondrial reactive oxygen probe MitoSOX (Invitrogen, Waltham, MA, USA). The fluorescence signals were measured by a flow cytometer (CyFlow Cube 6, Sysmex, Kobe, Japan).

### 4.7. Immunoblotting

Proteins were visualized by immunoblotting following the method we reported previously [61]. The protein bands transferred onto PVDF membranes were illuminated in an enhanced chemiluminescence reagent (SageCreation, Beijing, China) and detected with an imaging instrument (ChampChemi 910, SageCreation, Beijing, China). The images were semi-quantitatively analyzed using photoshop software (ver. CC 2015.5, Adobe, USA). The antibodies used in this study were ANXA1 (BS3438, Bioworld, Bloomington, MN, USA), LC3B (T55992, Abmart, Shanghai, China), p62 (18420-1-AP, ProteinTech, Rosemont, IL, USA), Tubulin α (ER130905, Huabio, Hangzhou, China) and HRP conjugated goat anti-rabbit (BS13278, Bioworld, Bloomington, MN, USA).

### 4.8. Clinical Sample and Animal Experiments

Tissues from 50 colon cancer patients were collected at No.903 Hospital of PLA Joint Logistic Support Force. This study obtained patients’ informed consent and was approved by the research ethics committee of No.903 Hospital of PLA Joint Logistic Support Force. Tumors and para-carcinoma tissues were paraffin-embedded and continuously sectioned for immunohistochemical assays, as previously described [61]. Mice experiments were performed according to the method of Talib et al. [63] and approved by the ethics committee of animal experiments at Zhejiang Sci-Tech University. For in vivo experiments, eighteen female BALB/c nude mice were randomly divided into two groups. Each mouse was subcutaneously injected with 7 × 10^6^ HCT116-control or HCT116-shANXA1 cells. Body weight and tumor volume were measured once every two days, beginning on the 12th day after injection and continuing for another 16 days. Then the tumors were isolated and weighed.

### 4.9. Statistical Analysis

All statistical data are presented as the means ± standard error of the mean (s.e.m.). The Student’s *t*-test was used for between-group analyses, and the log-rank test was used for survival analysis. *p* values of * < 0.05, ** < 0.01, and *** < 0.001 were considered significant.

## Data Availability

Data is contained within the article and Supplementary Material.

## Supplementary Materials

The following supporting information can be downloaded at: https://www.mdpi.com/article/10.3390/ph16010070/s1, Table S1: The information of 50 colon cancer patients.
